# Supplementary material for: Antibacterial immune functions of subadults and adults in a semelparous spider
Source: PeerJ. 2019 Oct 23;7:e7475. doi: 10.7717/peerj.7475 (PMC6815191; doi:10.7717/peerj.7475)
Supplement: Supplemental Information 1 [file peerj-07-7475-s001.zip › Data_analyses_report_V2.html]

Antibacterial immune functions of subadults and adults in a semelparous spider


# Antibacterial immune functions of subadults and adults in a semelparous spider

#### *Zoltán Rádai*

```
aa2 <- read.csv("ParagrOnt_Data_1_V1.csv", sep=",")
pp.asd <- read.csv("ParagrOnt_Data_2_V2.csv", sep=",")
```

### Summary of data

**Lab-reared spiders**

Variables:

- sampleID: unique identification number of each spider
- bgi: bacterial growth inhibition values (mean-centered, SD-scaled)
- cwl: cell wall lytic activity values (mean-centered, SD-scaled)
- stage: ontogenetic stage, subadults and adults as ***sa*** and ***a***, respectively
- sex: sex, females and males as ***F*** and ***M***, respectively
- ct.width.mm: prosoma width in mm
- petri: ID of petri dish in which samples from the given spider was used
- mom: ID number of the female spider from which spiders originated
- NO.bgi: boolean, indicating whether bgi was zero (1) or greater than zero (0)
- NO.cwl: boolean, indicating whether cwl was zero (1) or greater than zero (0)
- dev.time: number of days from hatching to the end of rearing
- growth.rate.3: growth rate as ***ct.width.mm***^3 divided by ***dev.time***

```
print(summary(aa2))
```

```
##     sampleID       bgi                cwl          stage   sex   
##  h1_2_13: 2   Min.   :-1.12908   Min.   :-1.7129   a :22   F:36  
##  h1_2_17: 2   1st Qu.:-0.47529   1st Qu.:-0.6853   sa:54   M:40  
##  h1_2_22: 2   Median : 0.05893   Median :-0.3503                 
##  h1_2_24: 2   Mean   : 0.06146   Mean   :-0.2568                 
##  h1_2_29: 2   3rd Qu.: 0.54904   3rd Qu.: 0.2189                 
##  h1_2_4 : 2   Max.   : 2.82922   Max.   : 1.9531                 
##  (Other):64                                                      
##   ct.width.mm        petri         mom         NO.bgi      
##  Min.   :1.388   b12    : 6   u3_2   :20   Min.   :0.0000  
##  1st Qu.:1.867   b16    : 6   u2_2   :16   1st Qu.:0.0000  
##  Median :1.944   b18    : 6   h1_2   :12   Median :0.0000  
##  Mean   :1.972   b4     : 6   h6_2   :12   Mean   :0.1974  
##  3rd Qu.:2.106   b9     : 6   h2_2   : 6   3rd Qu.:0.0000  
##  Max.   :2.395   b10    : 4   h10_2  : 4   Max.   :1.0000  
##                  (Other):42   (Other): 6                   
##      NO.cwl           dev.time     growth.rate.3    
##  Min.   :0.00000   Min.   : 66.0   Min.   :0.01735  
##  1st Qu.:0.00000   1st Qu.:120.0   1st Qu.:0.04868  
##  Median :0.00000   Median :149.0   Median :0.05988  
##  Mean   :0.07895   Mean   :136.7   Mean   :0.05946  
##  3rd Qu.:0.00000   3rd Qu.:156.0   3rd Qu.:0.07020  
##  Max.   :1.00000   Max.   :169.0   Max.   :0.10613  
##
```

**Spiders from natural habitat**

Variables:

- petri.ID: ID of petri dish in which samples from the given spider was used
- spider.ID: unique identification number of each spider
- status: reproductive status for a female being virgin or mated as ***F*** or ***F.cocoon***, respectively
- season: season group (i.e. time of collection), ***spring*** and ***autumn*** for early and late summer, respectively
- fecundity: number of offspring from the given female’s cocoon (if the female was mated)
- ct.width.mm: prosoma width in mm
- bgi: bacterial growth inhibition values (mean-centered, SD-scaled)
- cwl: cell wall lytic activity values (mean-centered, SD-scaled)

```
print(summary(pp.asd))
```

```
##     petri.ID       spider.ID        status       season      fecundity   
##  Min.   : 8.00   7-10   :  2   F       :120   autumn:102   Min.   :10.0  
##  1st Qu.:20.00   7-12   :  2   F.cocoon:188   spring:206   1st Qu.:22.0  
##  Median :42.00   7-16   :  2                               Median :33.0  
##  Mean   :38.45   7-17   :  2                               Mean   :30.7  
##  3rd Qu.:49.00   7-23   :  2                               3rd Qu.:38.0  
##  Max.   :65.00   7-28   :  2                               Max.   :60.0  
##                  (Other):296                               NA's   :202   
##   ct.width.mm         bgi                cwl          bgi.measurable  
##  Min.   :1.625   Min.   :-1.05936   Min.   :-1.3249   Min.   :0.0000  
##  1st Qu.:1.885   1st Qu.:-1.05936   1st Qu.:-1.3249   1st Qu.:0.0000  
##  Median :2.026   Median : 0.15261   Median :-0.1099   Median :1.0000  
##  Mean   :2.016   Mean   : 0.08756   Mean   :-0.2786   Mean   :0.7208  
##  3rd Qu.:2.135   3rd Qu.: 0.71679   3rd Qu.: 0.4558   3rd Qu.:1.0000  
##  Max.   :2.593   Max.   : 2.67574   Max.   : 3.1096   Max.   :1.0000  
##                                                                       
##  cwl.measurable  
##  Min.   :0.0000  
##  1st Qu.:0.0000  
##  Median :1.0000  
##  Mean   :0.6169  
##  3rd Qu.:1.0000  
##  Max.   :1.0000  
##
```

---

### Welch’s t-test

```
t.test(aa2$dev.time[aa2$stage=="sa"], aa2$dev.time[aa2$stage=="a"])
```

```
## 
##  Welch Two Sample t-test
## 
## data:  aa2$dev.time[aa2$stage == "sa"] and aa2$dev.time[aa2$stage == "a"]
## t = -6.2619, df = 64.102, p-value = 3.575e-08
## alternative hypothesis: true difference in means is not equal to 0
## 95 percent confidence interval:
##  -33.73488 -17.41664
## sample estimates:
## mean of x mean of y 
##  129.3333  154.9091
```

### Chi-squared test

```
chisq.test(table(aa2$stage, aa2$mom))
```

```
## Warning in chisq.test(table(aa2$stage, aa2$mom)): Chi-squared approximation
## may be incorrect
```

```
## 
##  Pearson's Chi-squared test
## 
## data:  table(aa2$stage, aa2$mom)
## X-squared = 9.7964, df = 8, p-value = 0.2796
```

### MCMCglmm models

***Note that estimates and P\_MCMC values will differ in each run of the model up to Monte-Carlo error.***

In the model output, stage-level estimates (e.g. ***traitbgi:stagesa***) are given for females, therefore the effect of sex (***M***) on its own can be interpreted as the sex difference, hence (unlike in stage differences) there is no need to contrast posterior distributions.

**Lab-reared spiders**

```
prmc1<-list(B=list(V=diag(8)*1e1, mu=rep(0,8)), 
            R=list(V=diag(2), nu=1), 
            G=list(G1=list(V=diag(2), nu=1), 
                   G2=list(V=diag(2), nu=1),
                   G3=list(V=diag(2), nu=1)))
asdmc.1<-MCMCglmm(cbind(bgi, cwl) ~ trait:stage + trait:sex + trait:growth.rate.3 - 1, 
                  random= ~ us(trait):sampleID + idh(trait):mom + idh(trait):petri, rcov=~us(trait):units, 
                  burnin=15000, nitt=115000, thin=20, prior=prmc1, family=rep("gaussian",2), data=aa2, verbose=FALSE)
```

```
## Warning: 'cBind' is deprecated.
##  Since R version 3.2.0, base's cbind() should work fine with S4 objects
```

```
print(summary(asdmc.1))
```

```
## 
##  Iterations = 15001:114981
##  Thinning interval  = 20
##  Sample size  = 5000 
## 
##  DIC: 134.5349 
## 
##  G-structure:  ~us(trait):sampleID
## 
##                            post.mean l-95% CI u-95% CI eff.samp
## traitbgi:traitbgi.sampleID   0.38712  0.16069   0.6628     5316
## traitcwl:traitbgi.sampleID  -0.03655 -0.20928   0.1370     5000
## traitbgi:traitcwl.sampleID  -0.03655 -0.20928   0.1370     5000
## traitcwl:traitcwl.sampleID   0.27227  0.09407   0.4852     5000
## 
##                ~idh(trait):mom
## 
##              post.mean l-95% CI u-95% CI eff.samp
## traitbgi.mom    0.6448  0.10079    1.570     4633
## traitcwl.mom    0.3656  0.04821    0.895     5000
## 
##                ~idh(trait):petri
## 
##                post.mean l-95% CI u-95% CI eff.samp
## traitbgi.petri    0.2463  0.06615   0.5100     5000
## traitcwl.petri    0.2455  0.06728   0.4897     5000
## 
##  R-structure:  ~us(trait):units
## 
##                         post.mean l-95% CI u-95% CI eff.samp
## traitbgi:traitbgi.units   0.07110  0.04290  0.10629     5000
## traitcwl:traitbgi.units   0.01017 -0.02474  0.04668     5000
## traitbgi:traitcwl.units   0.01017 -0.02474  0.04668     5000
## traitcwl:traitcwl.units   0.16567  0.09851  0.24376     5000
## 
##  Location effects: cbind(bgi, cwl) ~ trait:stage + trait:sex + trait:growth.rate.3 - 1 
## 
##                        post.mean l-95% CI u-95% CI eff.samp  pMCMC   
## traitbgi:stagea          0.15534 -0.74341  1.00980     4582 0.7120   
## traitcwl:stagea         -1.05263 -1.80186 -0.28267     5000 0.0096 **
## traitbgi:stagesa         0.43580 -0.38310  1.27642     5000 0.2872   
## traitcwl:stagesa         0.05639 -0.67175  0.76847     5000 0.8832   
## traitbgi:sexM           -0.49254 -1.03568  0.02644     5388 0.0680 . 
## traitcwl:sexM           -0.17238 -0.65136  0.35586     5000 0.4912   
## traitbgi:growth.rate.3   0.77068 -4.68392  6.80619     5000 0.7912   
## traitcwl:growth.rate.3   1.49546 -4.12330  7.00900     5000 0.6036   
## ---
## Signif. codes:  0 '***' 0.001 '**' 0.01 '*' 0.05 '.' 0.1 ' ' 1
```

```
# stage differences
bgi.dif.STAGE <- cbind(posterior.mode( asdmc.1$Sol[,"traitbgi:stagesa"]-asdmc.1$Sol[,"traitbgi:stagea"] ),
HPDinterval( asdmc.1$Sol[,"traitbgi:stagesa"]-asdmc.1$Sol[,"traitbgi:stagea"] ),
pMCMC( asdmc.1$Sol[,"traitbgi:stagesa"]-asdmc.1$Sol[,"traitbgi:stagea"] )[1,1] )
colnames(bgi.dif.STAGE) <- c("bgi", "HPDlower", "HPDupper", "PMCMC")

cwl.dif.STAGE <- cbind(posterior.mode( asdmc.1$Sol[,"traitcwl:stagesa"]-asdmc.1$Sol[,"traitcwl:stagea"] ),
HPDinterval( asdmc.1$Sol[,"traitcwl:stagesa"]-asdmc.1$Sol[,"traitcwl:stagea"] ),
pMCMC( asdmc.1$Sol[,"traitcwl:stagesa"]-asdmc.1$Sol[,"traitcwl:stagea"] )[1,1] )
colnames(cwl.dif.STAGE) <- c("cwl", "HPDlower", "HPDupper", "PMCMC")

print(bgi.dif.STAGE)
```

```
##           bgi   HPDlower  HPDupper  PMCMC
## var1 0.328831 -0.2928056 0.8490639 0.3136
```

```
print(cwl.dif.STAGE)
```

```
##           cwl  HPDlower HPDupper PMCMC
## var1 1.103488 0.6243457 1.665561 4e-04
```

```
# Accuracy of measures

##--- bgi ---
print(posterior.mode((asdmc.1$VCV[,"traitbgi:traitbgi.sampleID"])/(asdmc.1$VCV[,"traitbgi:traitbgi.sampleID"]+asdmc.1$VCV[,"traitbgi:traitbgi.units"])) )
```

```
##      var1 
## 0.8445612
```

```
print(HPDinterval( (asdmc.1$VCV[,"traitbgi:traitbgi.sampleID"])/(asdmc.1$VCV[,"traitbgi:traitbgi.sampleID"]+asdmc.1$VCV[,"traitbgi:traitbgi.units"]) ))
```

```
##          lower     upper
## var1 0.7177959 0.9381451
## attr(,"Probability")
## [1] 0.95
```

```
##--- cwl ---
print(posterior.mode((asdmc.1$VCV[,"traitcwl:traitcwl.sampleID"])/(asdmc.1$VCV[,"traitcwl:traitcwl.sampleID"]+asdmc.1$VCV[,"traitcwl:traitcwl.units"])) )
```

```
##      var1 
## 0.6409984
```

```
print(HPDinterval( (asdmc.1$VCV[,"traitcwl:traitcwl.sampleID"])/(asdmc.1$VCV[,"traitcwl:traitcwl.sampleID"]+asdmc.1$VCV[,"traitcwl:traitcwl.units"]) ))
```

```
##          lower     upper
## var1 0.3914933 0.7945658
## attr(,"Probability")
## [1] 0.95
```

```
#############################################################

# covariances

##--- G: random-effect ---
cov.G <- cbind(posterior.mode(asdmc.1$VCV[,"traitcwl:traitbgi.sampleID"]),
HPDinterval(asdmc.1$VCV[,"traitcwl:traitbgi.sampleID"]),
pMCMC(asdmc.1$VCV[,"traitcwl:traitbgi.sampleID"])[1,1] )
colnames(cov.G) <- c("Cov", "HPDlower", "HPDupper", "PMCMC")

##--- R: residuals ---
cov.R <- cbind(posterior.mode(asdmc.1$VCV[,"traitcwl:traitbgi.units"]),
HPDinterval(asdmc.1$VCV[,"traitcwl:traitbgi.units"]),
pMCMC(asdmc.1$VCV[,"traitcwl:traitbgi.units"])[1,1] )
colnames(cov.R) <- c("Cov", "HPDlower", "HPDupper", "PMCMC")

print(cov.G)
```

```
##              Cov   HPDlower  HPDupper PMCMC
## var1 -0.02099859 -0.2092833 0.1370136 0.654
```

```
print(cov.R)
```

```
##              Cov    HPDlower   HPDupper PMCMC
## var1 0.005290164 -0.02473948 0.04668004 0.564
```

---

**Spiders from natural habitat**

```
impr<-list(B=list(V=diag(8)*1e1, mu=rep(0,8)), 
           R=list(V=diag(2), nu=1), 
           G=list(G1=list(V=diag(2), nu=1), 
                  G2=list(V=diag(2), nu=1)))
immat.1<-MCMCglmm(cbind(bgi, cwl)~trait:status + trait:season + trait:status:season - 1, 
                  prior=impr, random=~us(trait):spider.ID + idh(trait):petri.ID, rcov=~us(trait):units, family=rep("gaussian", 2),
                  data=pp.asd, burnin=15000, nitt=115000, thin=20, verbose=FALSE)
print(summary(immat.1))
```

```
## 
##  Iterations = 15001:114981
##  Thinning interval  = 20
##  Sample size  = 5000 
## 
##  DIC: 726.1903 
## 
##  G-structure:  ~us(trait):spider.ID
## 
##                             post.mean l-95% CI u-95% CI eff.samp
## traitbgi:traitbgi.spider.ID   0.54008  0.40105   0.6800     5000
## traitcwl:traitbgi.spider.ID   0.01936 -0.09896   0.1368     5000
## traitbgi:traitcwl.spider.ID   0.01936 -0.09896   0.1368     5000
## traitcwl:traitcwl.spider.ID   0.68969  0.52002   0.8850     5000
## 
##                ~idh(trait):petri.ID
## 
##                   post.mean l-95% CI u-95% CI eff.samp
## traitbgi.petri.ID    0.1279  0.05024   0.2267     4457
## traitcwl.petri.ID    0.1641  0.06336   0.2970     5000
## 
##  R-structure:  ~us(trait):units
## 
##                         post.mean l-95% CI u-95% CI eff.samp
## traitbgi:traitbgi.units   0.11537  0.09157 0.142522     5350
## traitcwl:traitbgi.units  -0.01443 -0.03450 0.004969     5228
## traitbgi:traitcwl.units  -0.01443 -0.03450 0.004969     5228
## traitcwl:traitcwl.units   0.13594  0.10710 0.167335     5135
## 
##  Location effects: cbind(bgi, cwl) ~ trait:status + trait:season + trait:status:season - 1 
## 
##                                      post.mean  l-95% CI  u-95% CI
## traitbgi:statusF                      1.061363  0.663407  1.497055
## traitcwl:statusF                      0.448821 -0.008793  0.934361
## traitbgi:statusF.cocoon               0.430473  0.036545  0.804994
## traitcwl:statusF.cocoon              -0.796483 -1.241230 -0.376287
## traitbgi:seasonspring                -1.148238 -1.689108 -0.627473
## traitcwl:seasonspring                -0.768793 -1.374930 -0.147405
## traitbgi:statusF.cocoon:seasonspring  0.415943 -0.241982  1.118985
## traitcwl:statusF.cocoon:seasonspring  1.312469  0.543268  2.095251
##                                      eff.samp  pMCMC    
## traitbgi:statusF                         4801 <2e-04 ***
## traitcwl:statusF                         5000 0.0604 .  
## traitbgi:statusF.cocoon                  5000 0.0276 *  
## traitcwl:statusF.cocoon                  5306 0.0004 ***
## traitbgi:seasonspring                    5000 <2e-04 ***
## traitcwl:seasonspring                    5000 0.0148 *  
## traitbgi:statusF.cocoon:seasonspring     5000 0.2244    
## traitcwl:statusF.cocoon:seasonspring     5000 0.0008 ***
## ---
## Signif. codes:  0 '***' 0.001 '**' 0.01 '*' 0.05 '.' 0.1 ' ' 1
```

```
# contrasts between virgin and mated females
bgi.spring.dif <- (immat.1$Sol[,"traitbgi:statusF"] + immat.1$Sol[,"traitbgi:seasonspring"]) - 
  (immat.1$Sol[,"traitbgi:statusF.cocoon"] + immat.1$Sol[,"traitbgi:seasonspring"]) # interaction is not significant so not included
bgi.autumn.dif <- (immat.1$Sol[,"traitbgi:statusF"]) - 
  (immat.1$Sol[,"traitbgi:statusF.cocoon"])

cwl.spring.dif <- (immat.1$Sol[,"traitcwl:statusF"] + immat.1$Sol[,"traitcwl:seasonspring"]) - 
  (immat.1$Sol[,"traitcwl:statusF.cocoon"] + immat.1$Sol[,"traitcwl:seasonspring"] + immat.1$Sol[,"traitcwl:statusF.cocoon:seasonspring"])
cwl.autumn.dif <- (immat.1$Sol[,"traitcwl:statusF"]) - 
  (immat.1$Sol[,"traitcwl:statusF.cocoon"])

f.difs <- cbind(bgi.spring.dif, bgi.autumn.dif, cwl.spring.dif, cwl.autumn.dif)

# contrasts
print(posterior.mode(as.mcmc(f.difs)))
```

```
## bgi.spring.dif bgi.autumn.dif cwl.spring.dif cwl.autumn.dif 
##     0.57604367     0.57604367    -0.08770514     1.22097755
```

```
# HPD intervals
print(HPDinterval(as.mcmc(f.difs)))
```

```
##                      lower     upper
## bgi.spring.dif  0.09912082 1.1666053
## bgi.autumn.dif  0.09912082 1.1666053
## cwl.spring.dif -0.56107273 0.4201603
## cwl.autumn.dif  0.63227519 1.8780240
## attr(,"Probability")
## [1] 0.95
```

```
# P_MCMC values
print(pMCMC(f.difs))
```

```
##    pMCMC      variables
## 1 0.0248 bgi.spring.dif
## 2 0.0248 bgi.autumn.dif
## 3 0.7756 cwl.spring.dif
## 4 0.0000 cwl.autumn.dif
```

```
#############################################################

# covariances

##--- G: random-effect ---
cov.G.2 <- cbind(posterior.mode(immat.1$VCV[,"traitcwl:traitbgi.spider.ID"]),
HPDinterval(immat.1$VCV[,"traitcwl:traitbgi.spider.ID"]),
pMCMC(immat.1$VCV[,"traitcwl:traitbgi.spider.ID"])[1,1] )
colnames(cov.G.2) <- c("Cov", "HPDlower", "HPDupper", "PMCMC")

##--- R: residuals ---
cov.R.2 <- cbind(posterior.mode(immat.1$VCV[,"traitcwl:traitbgi.units"]),
HPDinterval(immat.1$VCV[,"traitcwl:traitbgi.units"]),
pMCMC(immat.1$VCV[,"traitcwl:traitbgi.units"])[1,1] )
colnames(cov.R.2) <- c("Cov", "HPDlower", "HPDupper", "PMCMC")

print(cov.G.2)
```

```
##              Cov    HPDlower  HPDupper  PMCMC
## var1 0.005578509 -0.09896409 0.1368182 0.7296
```

```
print(cov.R.2)
```

```
##              Cov    HPDlower    HPDupper  PMCMC
## var1 -0.01142849 -0.03450387 0.004968631 0.1368
```

### Linear regression

```
pp.f <- na.omit(pp.asd)[c(1:53)*2,]
for(i in unique(pp.f$spider.ID)){
  pp.f$bgi[pp.f$spider.ID==i] <- mean(pp.asd$bgi[pp.asd$spider.ID==i])
}

fecm.1<-lm(fecundity ~ bgi + cwl + season + bgi:season + cwl:season, data = pp.f)
print(summary(fecm.1))
```

```
## 
## Call:
## lm(formula = fecundity ~ bgi + cwl + season + bgi:season + cwl:season, 
##     data = pp.f)
## 
## Residuals:
##     Min      1Q  Median      3Q     Max 
## -12.512  -5.252  -1.372   5.488  17.190 
## 
## Coefficients:
##                  Estimate Std. Error t value Pr(>|t|)    
## (Intercept)        23.629      2.146  11.012  1.3e-14 ***
## bgi                -3.738      1.711  -2.184  0.03396 *  
## cwl                -1.678      1.404  -1.195  0.23804    
## seasonspring       10.682      3.569   2.993  0.00439 ** 
## bgi:seasonspring   -3.439      3.403  -1.010  0.31749    
## cwl:seasonspring   -1.949      2.768  -0.704  0.48474    
## ---
## Signif. codes:  0 '***' 0.001 '**' 0.01 '*' 0.05 '.' 0.1 ' ' 1
## 
## Residual standard error: 7.914 on 47 degrees of freedom
## Multiple R-squared:  0.589,  Adjusted R-squared:  0.5453 
## F-statistic: 13.47 on 5 and 47 DF,  p-value: 3.673e-08
```

```
fecm.1.r1<-lm(fecundity ~ bgi + cwl + season, data = pp.f)
print(summary(fecm.1.r1))
```

```
## 
## Call:
## lm(formula = fecundity ~ bgi + cwl + season, data = pp.f)
## 
## Residuals:
##     Min      1Q  Median      3Q     Max 
## -17.202  -5.365  -1.463   5.186  18.454 
## 
## Coefficients:
##              Estimate Std. Error t value Pr(>|t|)    
## (Intercept)    23.804      1.866  12.756  < 2e-16 ***
## bgi            -4.359      1.371  -3.180  0.00256 ** 
## cwl            -1.806      1.121  -1.611  0.11367    
## seasonspring   12.679      2.349   5.397 1.96e-06 ***
## ---
## Signif. codes:  0 '***' 0.001 '**' 0.01 '*' 0.05 '.' 0.1 ' ' 1
## 
## Residual standard error: 7.853 on 49 degrees of freedom
## Multiple R-squared:  0.5782, Adjusted R-squared:  0.5523 
## F-statistic: 22.39 on 3 and 49 DF,  p-value: 2.863e-09
```

### Binomial generalized linear regression

```
fecm.2 <- glm(bgi.measurable ~ fecundity * season, data=pp.f, family = "binomial")
print(summary(fecm.2))
```

```
## 
## Call:
## glm(formula = bgi.measurable ~ fecundity * season, family = "binomial", 
##     data = pp.f)
## 
## Deviance Residuals: 
##     Min       1Q   Median       3Q      Max  
## -2.1739  -0.6129   0.5478   0.8189   1.4811  
## 
## Coefficients:
##                        Estimate Std. Error z value Pr(>|z|)   
## (Intercept)             5.07094    1.88947   2.684  0.00728 **
## fecundity              -0.14773    0.06728  -2.196  0.02811 * 
## seasonspring           -2.50277    2.83419  -0.883  0.37720   
## fecundity:seasonspring  0.10195    0.08466   1.204  0.22850   
## ---
## Signif. codes:  0 '***' 0.001 '**' 0.01 '*' 0.05 '.' 0.1 ' ' 1
## 
## (Dispersion parameter for binomial family taken to be 1)
## 
##     Null deviance: 61.20  on 52  degrees of freedom
## Residual deviance: 53.32  on 49  degrees of freedom
## AIC: 61.32
## 
## Number of Fisher Scoring iterations: 5
```

```
fecm.3 <- glm(cwl.measurable ~ fecundity * season, data=pp.f, family = "binomial")
print(summary(fecm.3))
```

```
## 
## Call:
## glm(formula = cwl.measurable ~ fecundity * season, family = "binomial", 
##     data = pp.f)
## 
## Deviance Residuals: 
##     Min       1Q   Median       3Q      Max  
## -1.4515  -0.9373  -0.5067   0.9757   2.0574  
## 
## Coefficients:
##                        Estimate Std. Error z value Pr(>|z|)  
## (Intercept)              3.4747     2.2308   1.558   0.1193  
## fecundity               -0.2375     0.1178  -2.017   0.0437 *
## seasonspring            -5.5953     2.9859  -1.874   0.0609 .
## fecundity:seasonspring   0.2811     0.1275   2.204   0.0275 *
## ---
## Signif. codes:  0 '***' 0.001 '**' 0.01 '*' 0.05 '.' 0.1 ' ' 1
## 
## (Dispersion parameter for binomial family taken to be 1)
## 
##     Null deviance: 64.920  on 52  degrees of freedom
## Residual deviance: 54.829  on 49  degrees of freedom
## AIC: 62.829
## 
## Number of Fisher Scoring iterations: 6
```
